# Novel Insights in the Application of Probiotic Yeast *Saccharomyces boulardii* in Dairy Products and Health Promotion

**DOI:** 10.3390/foods13182866

**Published:** 2024-09-10

**Authors:** Zorica Tomičić, Ljubiša Šarić, Ružica Tomičić

**Affiliations:** 1 Institute of Food Technology in Novi Sad, University of Novi Sad, Bulevar cara Lazara 1, 21000 Novi Sad, Serbia; ljubisa.saric@fins.uns.ac.rs; 2 Faculty of Technology, University of Novi Sad, Bulevar cara Lazara 1, 21000 Novi Sad, Serbia; ruzica.tomicic@yahoo.com

**Keywords:** *Saccharomyces cerevisiae* var. *boulardii*, functional foods, yoghurt, mechanisms of action, genetic engineering

## Abstract

Probiotic organisms are increasingly being incorporated into foods in order to develop products to prevent and reduce many diseases. *Saccharomyces boulardii*, a probiotic yeast with unique properties, such as viability over a wide pH range, antibiotic resistance, and the ability to reach a steady state, has an advantage over bacterial probiotics. The present review highlights the potential application of *S. boulardii* in functional fermented dairy products and the genetic engineering of this probiotic microorganism as a therapeutic agent for the treatment of various infectious diseases. It was found that probiotic yeast stimulates the growth of lactic acid bacteria in dairy products, creating favorable conditions and positively affecting the product’s sensory characteristics. Moreover, its viability of more than 10^6^ cfu/mL at the end of the yogurt shelf life confirms its probiotic effect. On the other hand, there is a growing interest in the design of probiotic strains to improve their characteristics and fill existing gaps in their spectrum of action such as the inhibition of some bacterial toxins, as well as anti-inflammatory and immunomodulatory effects. The strengthening of immune functions and effective therapies against various diseases by *S. boulardii* was confirmed. However, considering this yeast species’ potential, further research is necessary to accurately determine the functional properties in terms of incorporation into food matrices and from the aspect of health and well-being claims.

## 1. Introduction

The modern way of life, dominated by stress, as well as irregular and unbalanced nutrition, has significantly influenced the number of diseases related mainly to the digestive system and increased body weight. The number changes annually, and according to the WHO, obesity rates are increasing worldwide and reaching more than 1 billion people [1]. For this reason, the food industry has recognized the need to prevent and solve these problems by producing foods that have functional value and by enriching products with probiotic cultures that have a beneficial effect on health [2]. Many studies are being conducted due to the growing interest in probiotics, and various definitions are in use. Experts of the working group of the Food and Agriculture Organization of the United Nations and the World Health Organization define probiotics as “live microorganisms which when administered in adequate amounts confer a health benefit on the host” [3]. There are numerous safety and functionality criteria in the selection process for defining probiotic species [4,5]. Therefore, in order to test the probiotic activity, numerous clinical trials are carried out against gastric or enterovirulent bacterial pathogens that cause disease in humans, representing a major health problem [6]. The majority of probiotics used today include species of lactic acid bacteria (LAB) and bifidobacteria, while other bacteria such as *Bacillus* and *Escherichia coli* are also used. However, more attention has recently been paid to the study and application of yeasts with probiotic potential, where *Saccharomyces boulardii* is the most common and widely studied species [7,8,9].

The use of probiotics represents a therapeutic potential in maintaining human health and in the treatment of various intestinal disorders by enhancing beneficial components of the intestinal microbiota as shown by much scientific and clinical evidence [10]. Several mechanisms of probiotic action have been described, and the most common are related to their ability to produce antimicrobial substances, compete with pathogens for adhesion to the epithelium and for nutrients, modulate the host’s immune system, and inhibit the production of bacterial toxin [5,11]. Although numerous studies in the field of probiotic bacteria such as live biotherapeutics are significant, comparably fewer attempts have been made in the genetic engineering of the probiotic yeast *S. boulardii* and its applications in the production of fermented milk products for similar purposes. The recent achievements and application of biotechnology in the dairy industry have enabled the development of quality characteristics of dairy products with additional enrichment with probiotics. As a functional food ingredient in the form of drugs or in food, *S. boulardii* has a beneficial effect on the human body and increases the biological activity of the normal intestinal microbiota [11,12]. Many desirable characteristics, such as increased resistance to acidic pH and the inherent ability to fold complex proteins and create post-translational modifications that might enable expression of a wide variety of therapeutic proteins in their proper conformation, make it a suitable live biotherapeutic vessel for application in the treatment of gastrointestinal disorders. *S. boulardii* is considered safe for consumption and has Generally Regarded As Safe (GRAS) status. Furthermore, the similarity between *S. boulardii* and well-characterized *Sacharomyces cerevisiae* is another advantage because engineering methods for *S. cerevisiae* are well established and allow for their application to *S. boulardii* as well [13,14]. This probiotic yeast can also stay longer in the gut than *S. cerevisiae*, making it a suitable host for metabolic engineering to produce biomolecules of interest in the gut [15].

The dairy industry can greatly benefit from new advances and innovations in modern biotechnology. The use of fermented dairy products in food as a source of probiotic microorganisms with therapeutic effect, rDNA technology, and improved tools for bioprocess engineering by producing new foods adapted to specific consumers is a very new and promising idea. Furthermore, one of the tendencies in medicine today is the ability to manipulate the genome as a tool in the fight against disease. Therefore, the aim of this review was to analyze health-promoting properties of *S. boualrdii* as designer probiotic strains and its potential application in functional dairy products.

## 2. Probiotic Yeast *S. boulardii* in Dairy Products

**Figure 1:**
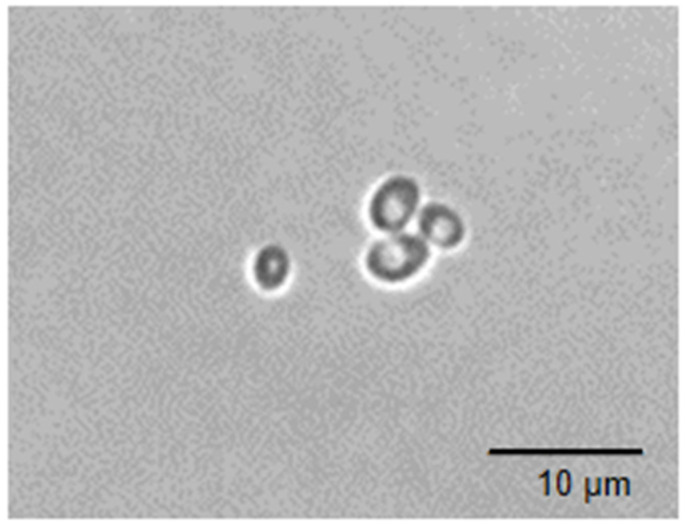
Image of *S. boulardii* cells taken using a microscope with a camera (Leica DFC290, Wetzlar, Germany) at a magnification of ×100.

Numerous researchers and certain sectors of the food industry have focused on the development of new food products where in-depth research is carried out on the specific properties of probiotics that not only have nutritional value for the consumer but also contribute to health, most of which relate to the health of the gastrointestinal tract, the immune system, and cancer. LAB and bifidobacteria are the most common bacterial strains of probiotics in the dairy industry today [12]. However, in recent decades, increased attention has been paid to eukaryotic microorganisms with probiotic properties to develop health-promoting products, where one of the most studied yeast strains is *S. boulardii* or *Saccharomyces* var. *boulardii* isolated from lychee fruit in Indochina by the French scientist Henri Boulard in 1920 [11]. The image of *S. boulardii* cells is presented in Figure 1. The cell of yeast is round or elliptical and between 4 and 8 μm in size. This probiotic is naturally resistant to antibiotics and can assimilate and ferment carbohydrates [9].

Although numerous studies have confirmed the utilization of *S. boulardii* for health-promoting purposes [5,6,7,8,9,10,11], little information is available on the effects of its supplementation to food. Therefore, research on its application as a starting culture for the development of probiotic and functional products has recently been initiated. Dairy products, fruit juices, and fermented beverages are the most studied foods that can be fermented with this yeast [16,17,18,19]. The most common fermented dairy products with probiotic yeast include acidophilus milk, bifidus milk, cheese, kefir, and yogurt, as presented in Table 1 [20,21,22,23]. Probiotic yeast possesses many advantages such as an optimal growth temperature of 37 °C and high viability, making it a good option for incorporation into food matrices [16]. Its viability is required to be at high levels, maintaining cell counts exceeding 10^6^ cfu/mL, since being subjected to harsh environmental conditions, such as food processing and storage, reduces their sustainability. Many technologies are being developed to protect probiotic cells, among which microencapsulation is considered an effective and novel technique to improve the viability of probiotics both in food products and in the intestinal tract. The most common microencapsulation techniques used in the food industry for the protection of probiotic microorganisms are coacervation, extrusion, emulsification, spray drying, and lyophilization [24,25]. Microencapsulation means using different carrier materials to improve probiotics addition and increase viability during storage in various food matrices. Furthermore, they should not have adverse effects on a product’s shelf life and sensory properties [16,26], and cells should remain viable (10^6^–10^7^ CFU per g) during the expected shelf life of the probiotic product [27]. The spray-drying technique is a well-known economical, efficient, and long-term preservation method for encapsulating probiotic cultures along with different carrier materials. It is widely used in the food industry to avoid the risk of biological and chemical degradation of food materials. This process has some disadvantages such as the loss of viability of the probiotic cells during drying at high temperatures. These limitations can be reduced by adding protectants such as proteins, polysaccharides, sugars and their combinations, prebiotics, and soluble fibers in the medium before spray-drying [24,25]. The consumption of 5 × 10^9^ CFU *S. boulardii* is recommended by the World Gastrointestinal Organization for certain gastrointestinal disorders such as antibiotic-associated diarrhea, *Clostridium difficile* infection, acute diarrhea, traveler’s diarrhea, and *Helicobacter pylori* infection [28].

In recent years, there has been an increasing tendency towards the creation of probiotic-enriched and functional fermented milk products, among which yogurt is considered the best delivery systems for probiotics. Notwithstanding the fact that *S. boulardii* is uncharacteristic in fermented milk products and has no ability to ferment lactose, it can be useful as a secondary flora in dairy products after LAB transforms lactose into glucose and galactose or after the intentional addition of sugar. Previous studies have shown that *S. boulardii* can stimulate the growth of lactic acid bacteria during co-fermentation in dairy products, since the yeast can metabolize both lactic acid and other organic acids that affect acidity, creating favorable condition for LAB growth and stability during storage [16,29,30]. A similar study was conducted by Karaolis et al. [31] during the processing of goat yogurt, suggesting the supplementation of *S. boulardii* after the starter has fermented and coagulated the milk. The presence of yeast stimulated the survival of LAB, and the final product was more stable without significant changes in taste. This association also improves the antioxidant properties of fermented final products [16]. Among other things, probiotic yeast can increase riboflavin levels as well, which is an important component of cellular metabolism and is responsible for normal development, growth, reproduction, lactation, and physical performance of well-being [32,33].

The consumption of dairy products with prebiotics, such as inulin and oligofructose, is currently growing due to numerous health effects such as strengthening of immune functions, promoting the growth of *Lactobacillus* and *Bifdidobacteria* in the colon, and increasing antioxidant activity as well as the bioavailability of various minerals such as iron and calcium. It was found that the combination of probiotic yeast and prebiotic inulin can significantly affect the textural and sensory properties of symbiotic yogurt and has a positive effect on textural characteristics such as hardness, cohesiveness, and adhesiveness, while yeast alone reduced hardness [19]. Recently, Sarwar et al., 2022 [34], showed a significant increase in the concentration of volatile compounds in symbiotic yogurt made with the probiotic *S. boulardii* in combination with inulin, indicating that the yeast can ferment available sugars. In addition, this product with higher amounts of antioxidants and volatile compounds improves consumer acceptance. Inulin supplementation also maintained the survival of *S. boulardii* viable in yogurt with a count greater than 10^6^ cfu/mL, which is generally required for probiotics. On the other hand, ice cream as a symbiotic product can serve as a good vehicle for probiotic delivery to the human gastrointestinal tract. The population of *Lactobacillus acidophilus* and *S. boulardii* in the symbiotic and probiotic ice cream was above the therapeutic levels during processing and storage, and their combination had a pronounced effect on the gut of human volunteers by increasing the number [35,36]. Ice cream containing probiotics and fructo-oligosaccharides as a prebiotic was also found to improve the microbiota in the human gastrointestinal tract [35].

In another study, Lourens-Hattingh and Viljoen [20] confirmed good survival of the probiotic strain *S. boulardii* ATCC 74012 in standard and UHT yogurt during 29 days of storage, reaching a maximum count exceeding 10^7^ cfu/g and an increased population in fruit yogurt attributed to the higher availability of fermentable sugars as well as fructose and sucrose present in the fruit supplement. The addition of *S. boulardii* in experimental kefir significantly improved the taste and did not differ from kefir produced in the classical way, which indicates the potential application of this probiotic yeast in the industrial production of kefir [23]. Moreover, most probiotic products do not retain their probiotic properties until the end of their shelf life, where *S. boulrdii* appears as a promising alternative.

## 3. Advantages of Probiotic Yeast *S. boulardii*

**Figure 2:**
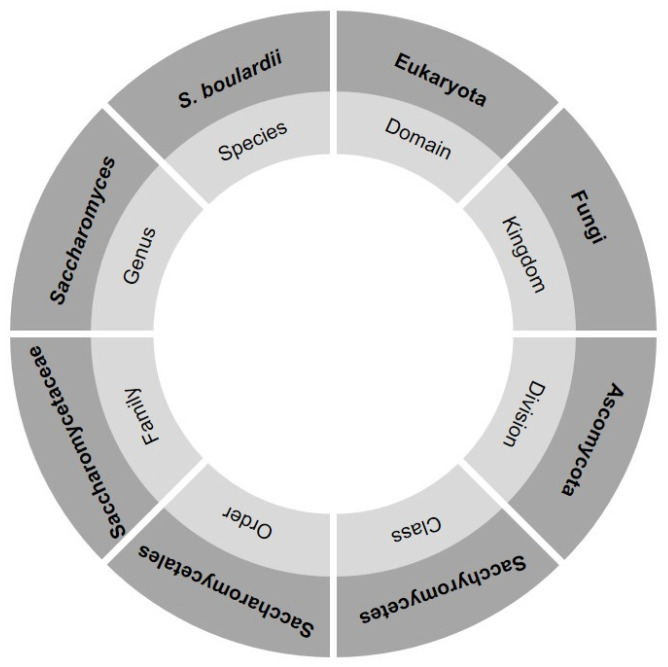
Current taxonomic position of *S. boulardii*.

Microorganisms intended for the purpose of probiotics must meet certain criteria in terms of growth, pH, salt resistance, and enzymatic activity, and they should have good adhesiveness to the intestinal mucosa. They also need to survive gastrointestinal transit and reach the site of action alive in order to exert their beneficial effects [8,16]. The current taxonomic affiliation of *S. boulardii* is shown in Figure 2. Despite some controversy about the taxonomic position, many molecular studies have indicated a close relatedness of *S. boulardii* to *S*. *cerevisiae* and, thus, support the recognition of *S. boulardii* as a member of *S. cerevisiae* and not as a separate species [9,37,38]. Excessive use of antibiotics leads to the emergence of antibiotic-resistant bacteria and the development of disorders of gut microbiota; therefore, the use of probiotic yeast has greater advantages compared to bacterial probiotics since it is resistant to antibiotics [17,39]. It is effective against a wide range of infections, such as inflammatory bowel disease, infections caused by *Helicobacter pylori*, and *Clostridium difficile* infections, and strong evidence has been found for its prevention and treatment of diarrheal diseases [9,17].

The mechanism of action of probiotics is related to their effect on the microbes that inhabit the gastrointestinal tract. However, the normal colonization of microbes in the human body is influenced by numerous factors, such as the immune system and the level of oxygenation and nutritional intake of the host’s tissues, so the mechanism of action of probiotics is very complex and strain-specific [6]. Recent studies have shown that probiotics can influence the normalization of the intestinal microbiota if it was previously disturbed by antibiotics and other stresses. Probiotic yeast interacts with intestinal bacteria upon entering the intestine, competes with pathogens for nutrients and suppresses their colonization, degrades *C. difficile* toxins A and B and their corresponding receptors on the colon mucosa, inhibits the production of bacterial toxins, maintains the integrity of the mucosal barrier, downregulates the production of inflammatory molecules (such as IL-8 and TNF-α) or increases the level of secretory IgA, and modulates the host’s immune system [40,41]. Another common mechanism of probiotic action is the production of diffusible antagonistic compounds that inhibit the growth of pathogenic bacteria [7]. *S. boulardii* produces many bioactive metabolites such as phenyllactic acid and 2-hydroxyisocaproic acid [42]. The study conducted by Tomičić et al. [8] reported the co-aggregation of *Candida* spp. with probiotic yeast *S. boulardii*. On the other hand, Gedek [43] observed the adhesion of Gram-negative enteropathogenic bacteria *Salmonella* Typhimurium and *Escherichia coli* to the surface of a probiotic yeast cell. These observations suggest that the adhesion of pathogenic microorganisms to the cell surface of *S. boulardii* may partially explain its protective effect. In addition, yeast cells are known to be rich in mannan, which supports the hypothesis that probiotic yeast exerts its effects by the trapping pathogenic bacteria on its surface, which may underlie many of the probiotic effects observed during infection [7,11].

*S. boulardii* has been widely studied against various disorders of the gastrointestinal tract due to its probiotic properties; however, few studies have been conducted on the use of this yeast as a vector for the expression of foreign genes of interest for biotechnological applications. Despite the similarity between *S. cerevisiae* and *S. boulardii*, not all genetic tools used in the genetic manipulation of *S. cerevisiae*, which is intensively used in biotechnological applications, can also be applied to *S. boulardii*; however, several genes are present. *S. boulardii* expresses greater resistance to higher temperatures and acidic pH [44]. DNA transformation into *S. boulardii* requires the selection of appropriate mutants that accept a suitable selection marker on the plasmid carrying the gene encoding that marker. Antibiotic resistance markers are widely used, but their clinical application is considered a major concern because of the transfer of antibiotic resistance to the human microbiome [45]. As a better alternative to antibiotic resistance, markers can be the transformation of diploid yeast with plasmids containing auxotrophic marker genes, as they are indigenous. However, the application of these selection markers requires appropriate host strains that are auxotrophic for the specific nutrient due to the absence of a functional chromosomal copy of the marker gene and can be propagated only in a medium containing the appropriate nutrient for growth. The most commonly used auxotrophic marker genes are wild-type alleles of yeast genes that encode key enzymes in metabolic pathways towards essential monomers used in biosynthesis [46], a typical example is the *URA3* gene [45]. In recent studies, uracil auxotrophic mutants of *S. boulardii* were generated using UV mutagenesis as hosts for recombinant protein production [45,47]. The results indicated that auxotrophic mutant strains of *S. boulardii* can express recombinant protein and are resistant in vitro to low pH, bile acid salts, and anaerobic conditions similar to those of the gastrointestinal tract, confirming their potential applications in the production and delivery of various recombinant products. However, the non-specific nature of UV mutagenesis can cause additional mutations in genes other than URA3 and lead to altered phenotypes associated with probiotic traits that have a negative impact on this yeast [15]. Thus, there is a need to develop auxotrophic *S. boulardii* mutants without the risk of causing other unwanted mutations in the genome and without the use of antibiotic resistance markers.

## 4. Applications of Designer *S. boulardii* for Health Benefits

Rapid advances in genome editing technologies have opened up new possibilities for the precise and safe genetic perturbations required for the engineering of probiotics in medical applications. Examples of engineered bacteria as living biotherapeutics are substantial, compared to the fewer attempts to engineer yeast for similar purposes [14]. Multiple favorable characteristics of *S. boulardii* have raised the interesting prospect of using this probiotic yeast as a vehicle for drug delivery to the intestine, such as increased resistance to bile salts, acidic pH, and higher temperatures [48]. These beneficial properties make *S. boulardii* a promising tool for oral vaccines, since orally administered antigens entering the gastrointestinal tract must be able to resist harsh digestive conditions, where the entering molecules are exposed and readily degradable in acidic environments and in the presence of digestive enzymes [37]. As a eukaryotic organism, *S. boulardii* is capable of creating post-translational modifications that might enable the expression of a broad array of therapeutic proteins in their proper conformation and the ability for antigens to be glycosylated as needed in the eukaryotic immune system [47].

**Figure 3:**
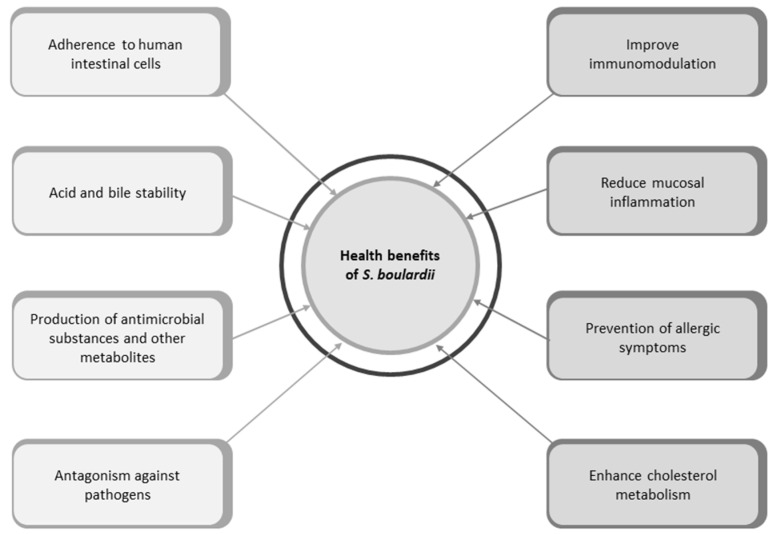
Health advantages of probiotic yeast *S. boulardii*.

*S. boulardii* is a probiotic yeast that has been proven effective in the context of many infectious and inflammatory gastrointestinal disorders. It has a wide range of beneficial effects against bacterial infections: degrading *C. difficile* toxins A and B and their respective receptors on the colon mucosa, inhibiting the actions of cholera toxins, modulating the immune system and the transduction pathway when activated by enteropathogenic bacteria, downregulating the production of inflammatory molecules such as IL-8 and TNF-α, and increasing secretory IgA levels [7,44]. The health advantages of probiotic yeast are demonstrated in Figure 3.

The CRISPR/Cas9 system is a recently developed genome-editing tool in yeast genomic engineering compared to traditional methods, making it favorable for developing auxotrophic mutations in *S. boulardii*. Relatively precise, efficient, flexible targeting, and marker-free features are the main advantages of the CRISPR/Cas9 technology for yeast genome engineering [15,49]. The CRISPR/Cas9 system uses a specific CRISPR guide RNA (gRNA) to guide the Cas9 endonuclease to cut designated gene targets and cause a double-strand break (DSB). Such DSBs can be repaired mainly through either the more predominant non-homologous end joining (NHEJ) that introduces a loss-of-function mutation through a reading frame shift or a premature stop codon, or a directed homologous recombination (HR) process that integrates transgene sequences into the genomic DNA through a supplied repair template [50]. The efficiencies of genetic perturbations are as high as 100%, and targeted genetic perturbations occur due to precise cutting and almost no off-target effects of the CRISPR/Cas9 system in yeast. Furthermore, the CRISPR/Cas9 system does not leave scars or unnecessary genetic elements in the genome, while other existing genetic perturbation methods do [15,51].

### 4.1. Targetting Viral and Bacterial Infections

Advances in genetic engineering have made yeasts valuable in the production of recombinant therapeutic proteins and subunit vaccines as they are considered as safe and effective due to their non-infectiveness and high immunogenicity. In the last few decades, yeasts have occupied a special place in the development of subunit vaccine formulations containing protein antigens that are synthesized in heterologous host cells against a wide range of diseases caused by bacteria and viruses. Recombinant hepatitis B virus (HBV) and human papillomavirus (HPV) subunit vaccines were the first approved for human use, and in both cases, the recombinant protein forms highly immunogenic virus-like particles that have been found to be more stable and significantly more immunogenic than purified protein antigens [52,53]. As the number of new infections caused by the human immunodeficiency virus (HIV) increases by about 2 million every year [54], antiretroviral therapy is not sufficient to completely eradicate the virus from the organism. Therefore, novel approaches are needed for the development of HIV vaccines. The use of genetically engineered probiotic *S. boulardii* expressing the HIV Gag antigen on its surface has shown promising results in preclinical studies [55], which encourages its application in stimulating immune responses against HIV in the mucosa.

On the other hand, in a recent study, *S. boulardii* was used for the first time in the production of an oral vaccine against the globally important pathogenic bacteria *Vibrio cholerae*, and it proved to be a promising host for the expression of *Vibrio cholerae* TcpA toxin [56]. Furthermore, research conducted by Liu et al. [16] demonstrated that *S. boulardii* engineered by CRISPR/Cas9 technology possess the ability to secrete a beneficial compound for human gut health such as human lysozyme by the integration of the human lysozyme gene (cHLY) with the chicken secretion signal into the genome of *S. boulardii*. Human lysozyme is a glycoside hydrolase that catalyzes the hydrolysis of 1,4 glycosidic linkages between the N-acetylmuramic acid and N-acetylglucosamine groups that occur in the peptidoglycan cell wall structure of certain microorganisms, particularly Gram-positive bacteria, and threatens the integrity of bacterial cell walls causing bacterial lysis [15].

### 4.2. Targetting Inflammation and Immune Modulation

It has been shown that probiotic *S. boulardii* induces different types of immune responses and possesses the ability to limit inflammation and infection in the gastrointestinal tract. Its ability to express and deliver recombinant proteins through the harsh digestive conditions of the intestine is the main requirement for the application of probiotic yeast as drug-delivery vehicles [18]. In the case of cytokine delivery, the ability of the probiotic to contact the immune tissues of the small intestine is crucial. Probiotic yeast *S. boulardii* induces anti-inflammatory responses by producing the anti-inflammatory cytokine interleukin 10 (IL-10) to immune tissues of the gastrointestinal tract [44,47]. The introduction of transformed yeast into Peyer’s patches, which are the major sites of antigen sampling from the small intestinal lumen as well as key sites of the induction and development of the immune response, would indicate the ability of yeast to survive passage through the gastrointestinal tract and contact tissues responsible for mediating immune responses [55]. In a study conducted by Hudson et al. [47], high levels of GFP expression in transformed *S. boulardii* Mutant 2 recovered from Peyer’s patches indicated that this mutant is resistant to a low pH and bile salt and possess the ability to deliver recombinant protein to the gastrointestinal immune tissues. Recently, Bagherpour et al. [48] successfully applied an OVA-producing recombinant *S. boulardii* strain to bring a highly immunogenic protein, ovalbumin, into the intestinal lumen via mice oral administration and observed a significant increase in IgA and IgG responses in the treated group compared to the control groups.

The ability of engineered *S. boulardii* to stimulate the immune system by increasing antibody levels, together with other probiotic properties, makes it a potential carrier for the oral delivery of immunogenic or therapeutic proteins into the intestinal lumen, implying its administration to the healthy host mucosa. However, there is a little information on the interaction between *S. boulardii* and the mucosal immune system in a healthy intestine compared to an inflamed mucosal tissue. A diverse microbiota, an intact epithelial barrier, and fewer inflammatory immune cells in the healthy gastrointestinal tract may limit the extent to which *S. boulardii* comes in contact with and affects the host’s mucosal immune system. Hence, understanding the interactions between probiotics and healthy mucosa is crucial for the application of *S. boulardii* in oral vaccine delivery or prophylaxis [16,48]. A previous study indicated limited immunomodulatory properties of the probiotic yeast *S. boulardii* in the healthy intestine and a little effect in potentiating B and T cell responses, possibly due, in part, to limited contact between probiotics and immune cells [16]. Similar studies using different engineered yeasts have also shown promising results for the application of “Whole Yeast Vaccine” as a new platform for vaccine development, especially for oral immunization strategies [53,57].

### 4.3. Targetting Cancer

The immunomodulatory properties of β-glucans present in the yeast cell wall have encouraged the application of yeast for the development of therapeutic cancer vaccine formulations. β-glucan stimulates the immune system by enhancing antitumor cytotoxic T-cell responses and the possibility of the intracellular expression of heterologous tumor antigens. Its utilization as “adjuvant therapy” in cancer therapy has been disclosed in patent filings due to its positive effects and the enhanced effectiveness of anticancer agents in their combination [53,57]. In order to make yeast-based therapeutic vaccine effective against cancer, the yeast must be engineered to express tumor-specific antigens, selectively expressed or overexpressed on malignant cells based on several criteria [52,56]. Numerous tumor antigens are currently being investigated in preclinical and clinical studies. An example is the mutated Ras protein, selected as the ideal target antigen for yeast-based immunotherapy due to its important role as a trigger of tumorigenesis in several types of human cancer [58]. Fu et al. [42] investigated the metabolites 2-hydroxyisocaproic acid and phenyl lactic released by *S. boulardii* and found that it exhibits strong anticancer properties. In another study, metabolites of probiotic yeast significantly inhibited the expression of IL-8 and NFƙB genes in colon cancer cells, suggesting their potential application in the prevention and treatment of colorectal cancer in humans [59,60]. Furthermore, various bioactive metabolic compounds such as polyphenols, peptides, organic acids, and short-chain fatty acids released and produced by probiotics showed antioxidant and anticancer properties.

## 5. Conclusions

*S. boulardii* is a unique probiotic and biotherapeutic yeast that has been successfully used in the treatment of various digestive disorders in humans. Due to the beneficial effects attributed to this probiotic yeast, scientific interest in its application in food with functional characteristics has increased. Recent research indicates that the *S. boulardii* yeast has the ability to stabilize the pH and acidity of food matrices for a longer period of time and, thus, provides an ideal environment for the growth of lactic acid bacteria in dairy products. It is also stable under food storage conditions and has a positive effect on the sensory quality of dairy products. Moreover, *S. boulardii* has a good ability to survive in cow’s milk yogurt and in kefir in numbers higher than 10^6^ cfu/mL, which is required for its probiotic properties.

On the other hand, interest in the genetic engineering of probiotic microorganisms as therapeutic agents for the treatment of various infectious diseases has increased significantly in recent decades. Probiotic yeast has confirmed immunomodulatory effects and effective therapies against various diseases. Numerous studies have shown that *S. boulardii* produces a wide range of bioactive metabolites that have antibacterial, antioxidant, antitumor, and anti-inflammatory properties. Therefore, there is a great interest in those strains that have a proven probiotic potential to be designed to improve their useful characteristics and fill existing gaps in their spectrum of activity as a probiotic for their safer and more effective application in humans.

## Figures and Tables

**Table 1:** Fermented dairy products with different strains of probiotics.

Product Name	Probiotic Strain	Reference
Acidophilus milk	*L. acidophilus; Sac. boulardii*	Lourens-Hattingh and Viljoen, 2001 [20].
Bifidus milk	*B. longum; B. bifidum; Sac. boulardii*	Wang and Feng, 2019 [21].
Cheese	*Lc. Lactis; S. thermophiles; Sac. boulardii*	Zamora, 2013 [22].
Kefir	*L. casei; L. acidophilus; L. paracasei; L. fermentum; K. marxianus; S. unisporus; Sac. cerevisiae; Sac. boulardii*	Ivanova, 2012 [23].
Yogurt	*L. acidophilus; L. bulgaricus; L. rhamnosus; L. plantarum; L. helveticus; L. casei; L. fermentum; S. thermophilus; B. lactis; Sac. boulardii*	Lourens-Hattingh and Viljoen, 2001 [20].

*L., Lactobacillus; Lc., Lactococcus; S., Streptococcus; B., Bifidobacterium; K., Kluyveromyces; Sac., Saccharomyces.*

## Data Availability

No new data were created or analyzed in this study. Data sharing is not applicable to this article.
